# Post‐Degradation Recovery of CsPbI_3_ Quantum Dot Solar Cells

**DOI:** 10.1002/smll.202409709

**Published:** 2025-01-09

**Authors:** Julius Brunner, Angelika Wrzesińska‐Lashkova, Lucas Scalon, Ruth Pinheiro Muniz, Anatol Prudnikau, Darius Pohl, Markus Löffler, Fabian Paulus, Yana Vaynzof

**Affiliations:** ^1^ Chair for Emerging Electronic Technologies TUD Dresden University of Technology Nöthnitzer Straße 61 01187 Dresden Germany; ^2^ Leibniz Institute for Solid State and Materials Research Dresden Helmholtzstraße 20 01069 Dresden Germany; ^3^ Institute of Chemistry University of Campinas (UNICAMP) São Paulo 13083–970 Campinas Brazil; ^4^ Dresden Center for Nanoanalysis (DCN) Center for Advancing Electronics Dresden (cfaed) TUD Dresden University of Technology Helmholtzstraße 18 01069 Dresden Germany; ^5^ Center for Advancing Electronics Dresden (cfaed) Helmholtzstraße 18 01069 Dresden Germany

**Keywords:** CsPbI_3_, degradation, perovskite nanocrystals, quantum dot photovoltaics, solar cell stability, surface passivation

## Abstract

The stability of perovskite quantum dot solar cells is one of the key challenges of this technology. This study reveals the unique degradation behavior of cesium lead triiodide (CsPbI_3_) quantum dot solar cells. For the first time, it is shown that the oxygen‐induced degradation and performance loss of CsPbI_3_ quantum dot photovoltaic devices can be reversed by exposing the degraded samples to humidity, allowing the performance to recover and even surpass the initial performance. By careful characterization and analysis throughout the degradation and recovery process, the underlying physical and chemical mechanisms that govern the evolution of the device performance could be identified. It is shown that the ligand shell of the quantum dots, rather than the instability of the semiconducting material itself, is the driving factor in these mechanisms. This highlights the important role of surface chemistry and ligand design in enhancing perovskite quantum dot photovoltaics.

## Graphical abstract

This study investigates the degradation and recovery behavior of CsPbI_3_ quantum dot solar cells. For the first time, it reveals that oxygen‐induced performance loss can be reversed by exposing the devices to humidity, leading to improved performance. The findings highlight the critical role of surface chemistry and ligand design in stabilizing CsPbI_3_ quantum dots for photovoltaics.

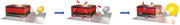

## 1 Introduction

Metal halide perovskites have attracted immense scientific attention over the past decade due to their remarkable optoelectronic properties and potential for applications in various optoelectronic devices.^[^ ^1^, ^2^, ^3^, ^4^, ^5^ ^]^ Among the various perovskite compositions, fully inorganic lead halide perovskite quantum dots (QDs), such as cesium lead triiodide (CsPbI_3_) QDs, are known for their advantageous optoelectronic properties such as high absorption coefficients, exceptionally high photoluminescence quantum yields, multiple exciton generation, high defect tolerance, and improved phase stability under harsh environmental conditions.^[^ ^6^, ^7^, ^8^, ^9^ ^]^ The inorganic nature of CsPbI_3_ makes it less sensitive to elevated temperatures and decomposition.^[^ ^10^ ^]^ However, the transformation into the non‐photoactive phase (δ‐phase) is considered to be the main driver for the degradation of fully inorganic perovskites.^[^ ^11^ ^]^ This is because of the relatively small ionic radius of Cs, resulting in a small Goldschmidt tolerance factor of about 0.822.^[^ ^12^ ^]^ The α crystal phase of CsPbI_3_ is only stable at temperatures above 350 °C and converts to the δ‐phase at room temperature.^[^ ^13^ ^]^

Reducing the dimensionality of CsPbI_3_ from bulk to QDs improves the phase stability tremendously, first discovered in 2015 by Protesescu et al.^[^ ^6^ ^]^ The QD structure provides confinement and surface passivation by ligands, enhancing the stability against environmental factors. Moreover, the size of the QDs can be easily tuned by varying the synthesis temperature, allowing for precise control over their absorption properties. This tunability makes CsPbI_3_ QDs promising candidates for tandem solar cell applications, where the absorption must be matched to the respective sub‐cell. In 2016, Swarnkarn et al., for the first time, applied CsPbI_3_ QDs in photovoltaics and LEDs with a maximum power conversion efficiency (PCE) of 10.77%.^[^ ^14^ ^]^ Since then, the efficiency has further increased, surpassing 16% at present.^[^ ^15^, ^16^, ^17^, ^18^, ^19^ ^]^ These advances were made possible by developing novel passivation strategies to enhance the stability and inter‐dot coupling of CsPbI_3_ QDs. For instance, formamidinium iodide,^[^ ^20^ ^]^ 2‐phenylethlyammonium iodide (PEAI),^[^ ^21^ ^]^ guanidinium thiocyanate,^[^ ^22^ ^]^ and choline iodide^[^ ^16^ ^]^ were successfully utilized for ligand exchange, leading to an enhancement in the photovoltaic performance but also the stability of the devices.

Despite these advances in stability, CsPbI_3_ quantum dot solar cells (QDSCs) are still susceptible to degradation upon exposure to oxygen, humidity, and light.^[^ ^23^ ^]^ To unveil the full potential of CsPbI_3_ QDSCs, it is necessary to comprehensively understand the devices’ degradation pathways and mechanisms. Recent studies in the field of CsPbI_3_ QDSC reported controversial results regarding the primary degradation mechanism, i.e., it remains unclear if water or oxygen are the main drivers for device degradation.^[^ ^24^, ^25^ ^]^

In this study, we investigate the degradation mechanisms of CsPbI_3_ QDSCs and analyze their origins. We show that the solar cell performance deteriorates under illumination in a dry oxygen environment but can be restored when exposed to a humid atmosphere. To the best of our knowledge, this humidity‐assisted recovery effect has yet to be documented for CsPbI_3_ QDSCs. We demonstrate that the humidity‐assisted performance recovery results in photovoltaic performances surpassing the initial device efficiencies. Our study reveals that the iodide‐based ligands and residual impurities in the CsPbI_3_ QD films are the source of this unique behavior. Our findings contribute to a better understanding of the degradation mechanism of all‐inorganic perovskite QDSC and are an initial step to enhance their stability and lifetime.

## 2 Results and Discussion

### 2.1 Quantum Dot Synthesis and Solar Cell Performance

**Figure 1:**
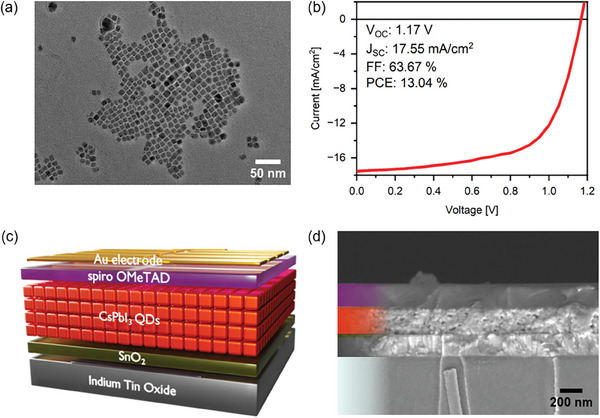
a) TEM image of CsPbI_3_ QDs. b) *J–V* curve of best performing QDSC. c) Scheme of a CsPbI_3_ QDSC. d) SEM cross‐section image of CsPbI_3_ solar cell device.

We synthesized CsPbI_3_ QDs via the hot‐injection synthesis first published by Protesescu et al. in 2015,^[^ ^6^ ^]^ with several modifications described in the experimental section to improve the yield and quality of the QDs. These QDs show a narrow size distribution and an average size of approximately 15 nm as determined by transmission electron microscopy (TEM), shown in **Figure** **1**a. The bandgap of approximately 1.77 eV makes the QDs a promising candidate for emerging photovoltaic applications (see Figure S1, Supporting Information).

After synthesizing and purifying the crude solution with methyl acetate, the QDs are stored as colloid ink in octane. Since the QDs are shelled with long‐chain organic ligands such as oleic acid and oleylamine, the dispersion remained stable even after several months of storage in ambient conditions, as reported already by others.^[^ ^14^ ^]^ To employ the QDs in solar cells, we exchanged the long‐chain ligands to allow for a sufficiently high inter‐dot conductivity in order to enable the transport and extraction of the photogenerated charges. The ligand exchange was achieved by a layer‐by‐layer (LbL) approach, first published by Svarnkar et al.^[^ ^14^ ^]^ For the LbL ligand exchange employed here, we exchanged the long‐chain organic ligands using sodium acetate and PEAI as ligands, following the method published by Jigeon Kim et al. in 2020.^[^ ^21^ ^]^ This method results in high‐performance QDSC with high resilience against moisture. Using a subsequent deposition of four layers of QDs, ≈250 nm thick films of CsPbI_3_ QDs were obtained. To allow charge extraction, the active layer was sandwiched between 30 nm of tin dioxide (SnO_2_) nanoparticles on one side as an electron extraction layer and 150 nm of doped 2,2′,7,7′‐tetrakis[N,N‐di(4‐methoxyphenyl)amino]‐9,9′‐spirobifluorene (spiro‐OMeTAD) on the other side as a hole transport layer (HTL). It is important to note that our devices were always exposed to an atmosphere of dry N_2_/O_2_ overnight before the evaporation of the top metal electrode to allow the oxidation of the HTL, according to the literature.^[^ ^26^ ^]^ Transparent patterned conductive indium tin oxide (ITO) and gold serve as cathode and anode, respectively. The schematic device structure is shown in Figure 1c,d shows a cross‐sectional scanning electron microscopy (SEM) image of the device stack. The performance of these QDSCs is comparable to the latest publications in this field,^[^ ^15^, ^16^, ^17^, ^18^, ^19^ ^]^ and the current density – voltage (*J–V*) characteristics of our champion solar cell and its photovoltaic parameters are shown in Figure 1b.

### 2.2 Photovoltaic Device Stability

To assess the stability of the CsPbI_3_ QDSCs, we exposed the devices to different surrounding atmospheres and systematically analyzed their performance throughout the exposure. In our study, we are able to separate the influence between oxygen‐ and humidity‐triggered degradation by exposing the devices and films to a precisely controlled atmosphere in a bespoke environmental chamber that allows an in‐situ characterization of the device performance in these conditions.^[^ ^27^, ^28^ ^]^ Parallel experiments in the same environmental chamber were conducted with the QD film itself without any extraction layer and electrode to facilitate spectroscopic characterization of the active layer.

**Figure 2:**
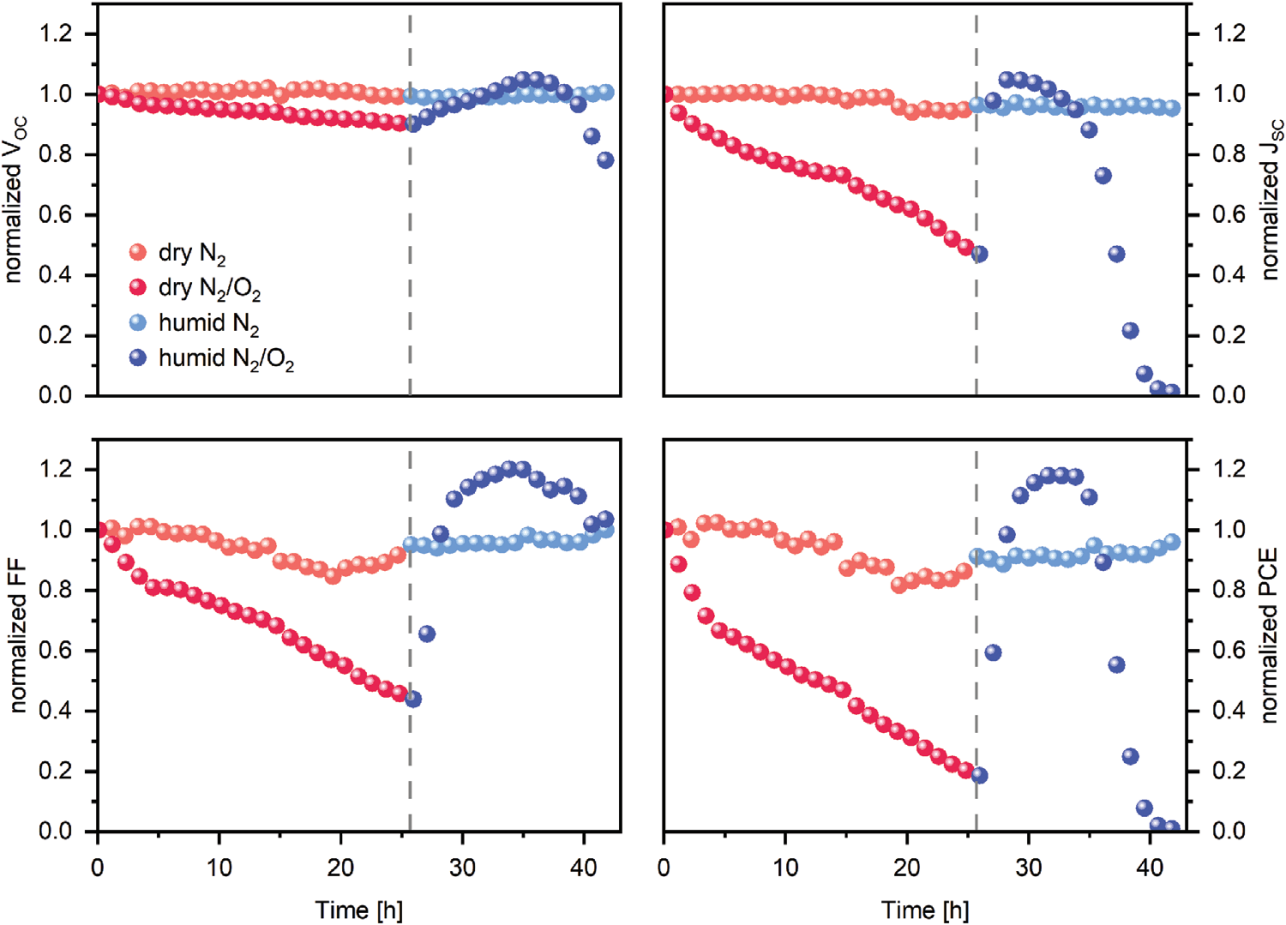
Normalized solar cell parameters during 42 h of degradation in different atmospheres. Dark colors show the evolution of the V_OC_, J_SC_, FF, and PCE in N_2_/O_2_, whereas light colors show the parameters of the reference experiment in the N_2_‐only atmosphere. Red corresponds to the respective dry atmospheres, and blue to the humid atmospheres. Dashed vertical reference lines indicate the change between dry and humid atmospheres.

We find that exposure to a dry mixed N_2_/O_2_ atmosphere (≈21% of oxygen) under continuous illumination leads to a fast PCE performance decay, as shown in **Figure** **2**. After 24 h of N_2_/O_2_ exposure, the performance of the QDSCs decreased to approximately 20% of their initial values. This oxygen‐induced degradation especially affects the short‐circuit current density (J_SC_) and fill factor (FF) of the devices, while the open‐circuit voltage (V_OC_) remains rather stable (Figure 2). This suggests that the QDs are still in their black phase and haven't converted to the yellow δ‐phase yet, as the δ‐phase is not photoactive, which consequently results typically in a dramatic loss in all solar cell parameters.^[^ ^11^ ^]^

After the samples were exposed to dry N_2_/O_2_, resulting in a significant loss in performance, we introduced 30% relative humidity into the environment and continued to track the QDSCs' photovoltaic parameters. Surprisingly, the performance dramatically and rapidly increased, even surpassing the initial performance within a few hours later. Although all photovoltaic parameters improve, it is the J_SC_ and FF that display a particularly abrupt increase upon the introduction of humidity. Although the performance recovery occurs within hours, it does not remain a lasting effect, and after prolonged exposure to the combination of oxygen and humidity, a pronounced drop in all photovoltaic parameters occurs due to the conversion of the CsPbI_3_ QDs into the non‐photoactive δ‐phase, as will be shown below. To untangle the influence of oxygen from that of the continuous illumination, we conducted the same experimental procedure with an N_2_‐only atmosphere, and we also introduced humidity after 26 h. Under these conditions, the QDSCs’ performance remained largely unchanged across the entire experiment, indicating that the presence of oxygen plays a critical role in the performance loss. We highlight that even the severe degradation in the humid atmosphere is not observed in the absence of oxygen, confirming that oxygen acts as the driving force for the performance degradation in CsPbI_3_ QDSCs, while humidity on its own does not. On the contrary, it seems to have a very beneficial effect on oxygen‐degraded samples.

**Figure 3:**
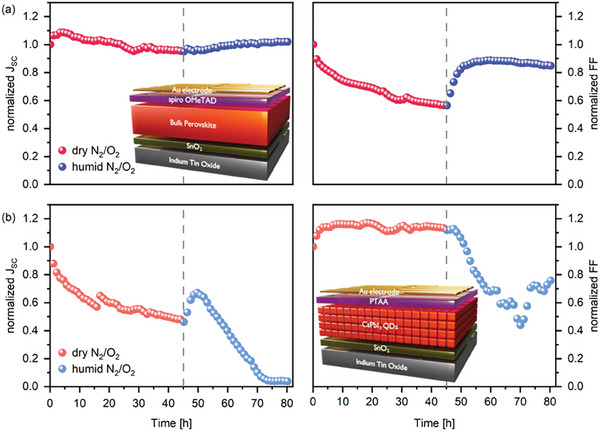
a) Evolution of J_SC_ and FF for bulk perovskite solar cells with the same architecture as CsPbI_3_ QDSCs. b) Evolution of J_SC_ and FF for CsPbI_3_ QDSC with doped PTAA instead of spiro‐OMeTAD HTL. Dashed vertical reference lines indicate the change between dry and humid atmospheres.

This peculiar degradation and recovery behavior of the CsPbI_3_ QDSCs could originate from different layers and interfaces within the solar cell stack. To investigate the underlying mechanism, we focused on the individual components of the multilayered QDSC during the degradation. To elucidate whether the active layer is the main source of this degradation behavior, we exchanged the active layer with bulk perovskite while keeping the other device layers the same.^[^ ^29^, ^30^ ^]^ **Figure** **3**a shows the evolution of the J_SC_ and FF for such devices. The J_SC_ remains largely stable for the dry N_2_/O_2_ atmosphere and consequently does not exhibit any degradation or recovery, unlike the case of CsPbI_3_ QDs. The FF, on the other hand, exhibits a clear deterioration upon dry exposure to the N_2_/O_2_ atmosphere, improving after introducing humidity into the atmosphere. Since both the bulk perovskite and the CsPbI_3_ QDSCs utilize spiro‐OMeTAD as HTL, we attribute the evolution of the FF to the impact of these environmental factors on the conductivity of this layer, as has been previously reported in the literature.^[^ ^26^, ^31^ ^]^

To further confirm that the decrease and subsequent improvement in FF observed for QDSCs is attributed to the use of spiro‐OMeTAD as HTL, we fabricated CsPbI_3_ QDSCs without spiro‐OMeTAD and utilized doped Poly‐[bis‐(4‐phenyl)(2,4,6‐trimethylphenyl)amine] (PTAA) as HTL, based on a previously published method (see Figure S2,Supporting Information).^[^ ^32^, ^33^, ^34^, ^35^ ^]^ The degradation behavior of PTAA‐based devices is largely opposite to that of the bulk perovskite with spiro‐OMeTAD (Figure 3b). The PTAA‐based devices experience a small increase of FF after the start of the degradation experiment – potentially attributed to an increase in the conductivity of PTAA through oxygen doping – followed by a plateau until the humidity is introduced when the FF decreases drastically. On the other hand, the J_SC_ of the PTAA‐based devices deteriorates similarly to the spiro‐OMeTAD‐based CsPbI_3_ QDSCs in the dry oxygen‐rich atmosphere and also exhibits an improvement once humidity is introduced. Taken together, these observations clearly show that the evolution of the FF is dictated by the spiro‐OMeTAD layer. At the same time, the decrease and subsequent increase in J_SC_ originate from the CsPbI_3_ QDs active layer.

### 2.3 Evolution of the Active Layer Properties Upon Exposure to Oxygen and Humidity

To investigate how exposure to oxygen and humidity impacts the properties of the QD active layer, thin films of CsPbI_3_ QDs were characterized at different time points. The first measurements were performed directly after fabrication without additional treatments, followed by a 24‐hour exposure to a dry oxygen atmosphere (termed pristine, 24 h dry, red color scheme). The following three measurements were performed after exposure to a humid environment for 1, 3, and 6 hours to examine the samples’ recovery process (termed + 1 h humid, + 3 h humid, + 6 h humid, blue color scheme).

**Figure 4:**
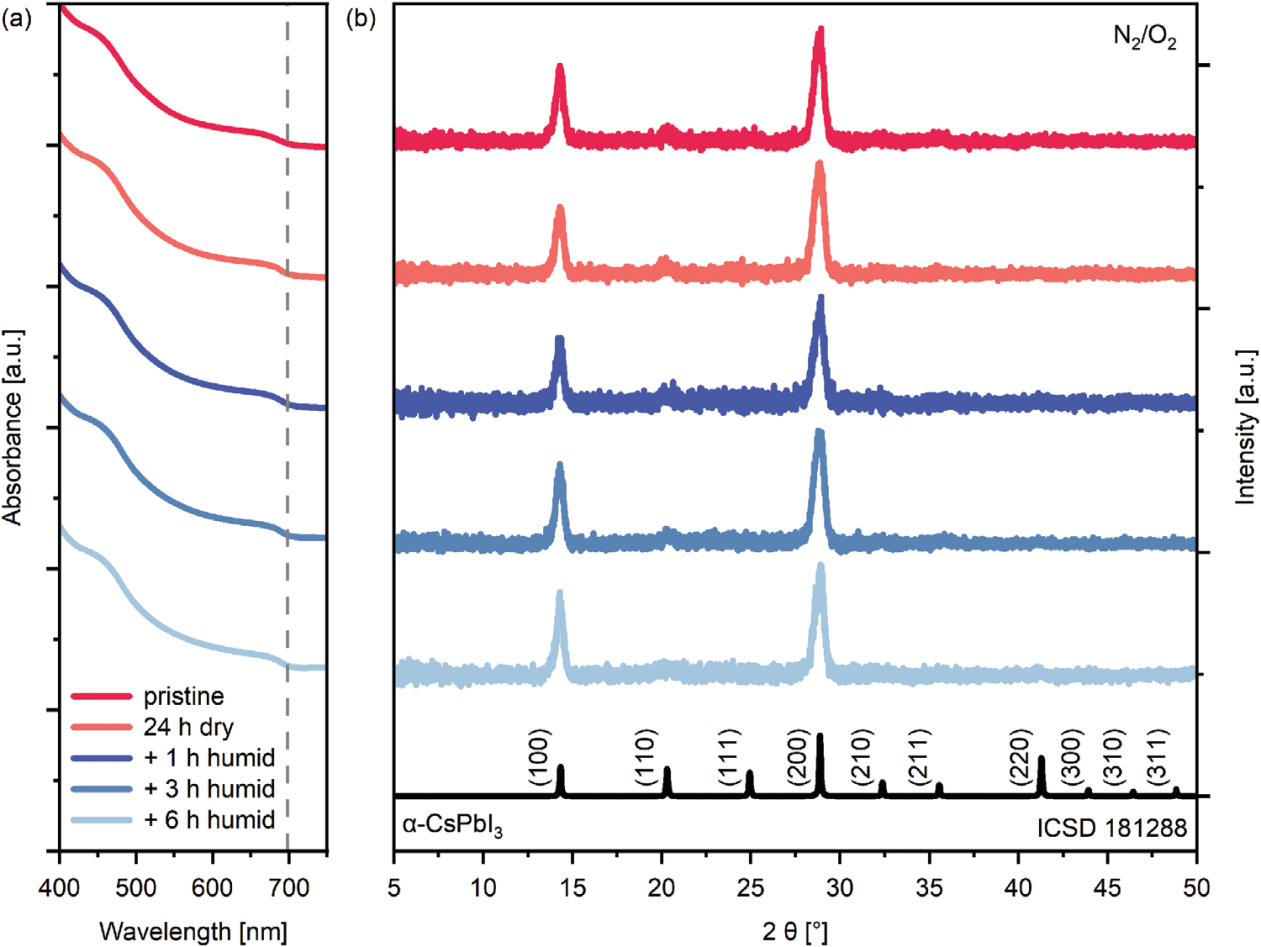
a) Staggered absorbance spectra of CsPbI_3_ QD layers after exposure to dry and humid N_2_/O_2_ atmosphere. The dashed line marks the bandgap calculated from Tauc plots (see Figure S1, Supporting Information). b) XRD pattern of the same QDs with theoretical data from the ICSD database.

**Figure** **4**a displays the optical absorbance spectra of the QD films in the N_2_/O_2_ atmosphere. For all measurements, the absorption edge of the QDs is at the same position of 698 nm (1.78 eV), and the shoulder (at approximately 675 nm) is of similar height, indicating that the active layers’ optical properties, as well as the bandgap and the QDs’ sizes, remain essentially unchanged throughout the degradation experiment. We characterized the films by X‐ray diffraction (XRD) to corroborate these findings. A comparison of the diffractograms to the theoretical data for cubic α‐CsPbI_3_ from the ICSD database (ICSD 181288) revealed that the QDs maintain the cubic phase throughout the experiment. In particular, we observe two strong reflections at 14.3° and 28.9°, corresponding to the (100) and (200) orientations of cubic CsPbI_3_, respectively. These measurements indicate the strong and near‐perfect orientation of the cubic QDs and the absence of impurity phases in our QDSCs. Moreover, no broadening of the reflections is observed, further confirming that the QDs’ sizes were not changed during the experiment. These measurements suggest that the performance evolution with an initial decrease in dry oxygen and recovery in a humid environment is not attributed to phase instabilities or a phase conversion of the perovskite QDs. Figure S3 (Supporting Information) shows the respective absorbance and XRD measurements for CsPbI_3_ QDs in a nitrogen atmosphere. Also in that case, neither the optical properties nor the phase purity change.

Next, we conducted XRD measurements at shallow incident angles of X‐rays (1° and 2° with respect to the sample's surface), which make it possible to investigate the QDs’ structure at the films’ surfaces (Figures S4 and S5, Supporting Information). The measurements show that close to the films’ surface, the QDs are not perfectly ordered, as reflections from other crystal orientations can be seen. However, despite this, we do not observe any deviations in the crystal phase or phase instabilities, confirming that the crystalline structure of the CsPbI_3_ QD active layer remains intact and is not the source of the performance degradation and humidity‐assisted recovery.

**Figure 5:**
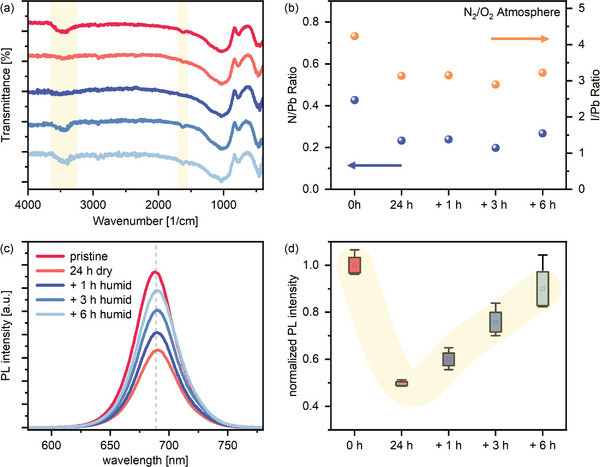
a) FTIR spectra of the treated samples in N_2_/O_2_. The region of the H‐O‐H bending mode at around 1700 cm^−1^ and O─H stretching at around 3500 cm^−1^ are highlighted in pale yellow. b) Evolution of the N/Pb and I/Pb ratios extracted from the XPS measurement of N_2_/O_2_ treated samples. c) Mean PL intensities of CsPbI_3_ films treated with dry and humid N_2_/O_2_. The dashed vertical reference line is located at 689 nm and serves as a guide for the eye. d) Statistical analysis of PL intensities with the box marking the standard deviation at each timestep, the whiskers marking the range of the measurement points, and the square marking the mean value.

Considering that the QD properties appear not to be affected by the environmental conditions, we turned our attention to the characterization of the QD ligand shell and the film composition. Fourier‐Transform Infrared Spectroscopy (FTIR) measurements were used to identify the presence of ligands in the QD film due to the individual vibrionic features of various molecules and substances used in the process. To increase the overall signal‐to‐noise ratio, we produced three QD films on glass that were exposed to the environmental conditions as described above. The films were then scratched off, compressed into a potassium bromide (KBr) pellet, and measured without further treatment (see experimental section for more information). This method is known to lead to low detection limits and high signal quality.^[^ ^36^ ^]^ **Figure** **5**a shows the FTIR spectra throughout the degradation and recovery experiment. The absence of features around 3000 cm^−1^ indicates that all the long‐chained aliphatic organic ligands, such as oleic acid and oleyl amine, have been successfully replaced in the fabrication of the films. While the vibrionic features between 500–1000 cm^−1^ remain largely unaffected throughout the experiment, differences at 1700 cm^−1^ and the broad band at 3500 cm^−1^ can be observed. Both bands are attributed to the presence of water with the H‐O‐H bending mode at around 1700 cm^−1^ and the symmetric and asymmetric O‐H stretching at around 3500 cm^−1^.^[^ ^37^ ^]^ These two signals indicate the presence of water molecules on the surface of the QDs or in the bulk of the film after fabrication. Upon light exposure to a dry N_2_/O_2_ atmosphere, the features attributed to water vanish within the first few hours, hinting at a complete removal of water from the QD film. A similar initial removal is observed for a dry nitrogen atmosphere (see Figure S6, Supporting Information). After introducing humidity into the system after 24 h, the characteristic vibrational features of water can only be detected for samples exposed to dry N_2_/O_2_ (see Figure 5a). In the case of dry nitrogen, no IR‐active bands for H_2_O appear, even after 6 h of exposure to high humidity levels (Figure S6, Supporting Information). This suggests that only in the case of samples exposed to an oxygenated atmosphere has the surface of the QDs been altered to favor the adsorption of water or hydroxyl moieties.

To investigate the evolution of the QDs’ composition, the samples were characterized by X‐ray photoemission spectroscopy (XPS). Figure 5b shows the evolution of the N/Pb and I/Pb ratio upon exposure to oxygen and, subsequently, humidity. The N/Pb ratio decreases from about 0.42 to 0.23, indicating the removal of PEAI ligands as the only N‐containing species after the LbL ligand exchange. In parallel, the overall iodide content in the films decreases during light and oxygen exposure: from an initial I/Pb ratio of ca. 4.2, the ratio decreases to a value very close to 3, as is expected from the ideal perovskite stoichiometry. Considering that the overall perovskite lattice is not affected in our experiments, the XPS measurements suggest that the films contain a substantial iodine excess following fabrication, which is eliminated from the film during 24 h of exposure to oxygen and light. This effect, however, does not occur for the QDs in the nitrogen atmosphere (see Figure S7a, Supporting Information). In this case, both the lead‐to‐iodine and the nitrogen‐to‐lead ratios remain stable at their initial values. We note that the cesium‐to‐lead ratios for both oxygen and non‐oxygen‐containing atmospheres maintain their initial ratio (see Figure S7b, Supporting Information), confirming that the composition of the QDs remains unaffected.

The layers were further characterized by photoluminescence (PL) spectroscopy to explore how changes in the film's composition impact the optoelectronic properties (Figure 5c). We observe that the emission wavelength at 689 nm remains unchanged throughout the experiments, further confirming that no changes to the perovskite core of the QDs’ size occur. Upon exposure to oxygen, the PL yield decreased markedly (Figure 5d), suggesting that new pathways for nonradiative recombination became available during this treatment. Upon exposure to humidity, the PL intensity recovers, approaching the original values after 6 h. These measurements suggest that a significant number of defects that serve as traps have formed during the exposure to oxygen and are later progressively eliminated in the early stages of exposure to humidity.

**Figure 6:**
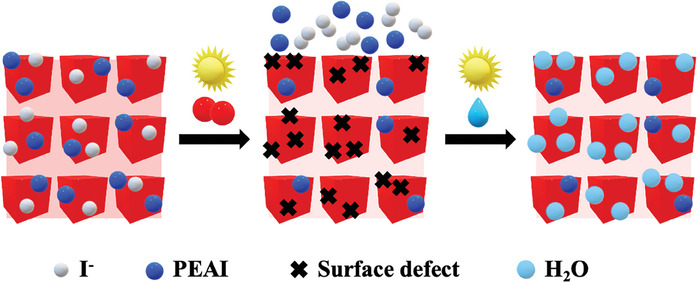
Schematic illustration of the proposed mechanism.

### 2.4 Proposed Mechanism for the Performance Evolution

The combination of these findings suggests that during the deterioration in the performance observed in oxygen and the subsequent recovery upon exposure to humidity, the perovskite core of the QDs remains intact. Instead, we propose that the surface ligands and the interdot‐media between the QDs play a major role in dictating the performance evolution of CsPbI_3_ QDSCs. The significant decrease in the I and N content is a consequence of photooxidative reactions that occur during the first 24 h of exposure to oxygen and light (**Figure** **6**). Iodide (I^−^) that passivates the surface of the QDs upon fabrication and is located in the interdot media is oxidized during this time period, forming potentially volatile iodine, which can leave the perovskite active layer. Oxygen can be localized on the formed iodine vacancies or adsorbed onto the QDs’ surface^[^ ^38^ ^]^ by capturing photoinduced electrons and forming superoxide or peroxide ions.^[^ ^39^, ^40^ ^]^ Both have a strong affinity for protons and can deprotonate ammonium cations, which passivate defects on the surface of the QDs. PEA^+^ can be deprotonated to form PEA^0^, leaving non‐passivated defects behind. PEA^0^ is liquid at room temperature and has a boiling point of 187 °C. Considering that the XPS measurements are performed in ultra‐high vacuum conditions, the PEA^0^ evaporates from the sample's surface, thus reducing the N/Pb ratio. The consequence of both these processes is the formation of non‐passivated defects at the QD surface, which agrees with the reduction of PL, V_OC,_ and J_SC_. Finally, upon exposure to humidity, water molecules or hydroxyl species passivate the defects formed during the first 24 h, enabling the recovery of the PL and the photovoltaic performance. The effect is transient, and upon subsequent exposure, the QDs’ cores are impacted and degrade by a phase transition to the δ‐phase (see Figure S8, Supporting Information).^[^ ^41^ ^]^ Interestingly, the effect can be repeated several times, with subsequent exposure to a dry oxygen/nitrogen environment leading to loss of performance, while recovery is possible with further exposure to humidity (Figure S9, Supporting Information), albeit with diminishing returns.

## 3 Conclusion

In this study, for the first time, we show the oxygen‐induced degradation and performance loss in CsPbI_3_ QDSCs, including the subsequent recovery. After careful characterization of CsPbI_3_ devices combined with the analysis of CsPbI_3_ films we were able to propose a physical‐chemical mechanism of this unique degradation behavior. According to this mechanism, the oxygen‐induced decrease in performance is a consequence of the de‐doping of spiro‐OMeTAD (leading to a reduced FF) and a loss in interdot I^−^ and PEAI that originally passivated the QDs (leading to a decrease in V_OC_ and J_SC_). This situation can be reversed by exposure to humidity, increasing the performance of the solar cells to levels that surpass the initial efficiency after several hours. The recovery can be attributed to the passivation of surface defects by water and hydroxyl groups. These findings provide important insights into the degradation mechanisms in QDSCs that are necessary for the development of mitigation strategies needed prior to the commercialization of this technology.

## 4 Experimental Section

### Materials

Cs‐carbonate (Cs_2_CO_3_; >98%), lead iodide (PbI_2_; 99.99%) were obtained from TCI. Oleic acid (OA; technical grade, 90%), oleylamine (OLA; technical grade, 70%), sodium acetate (NaOAc; 99.995%), 4‐tert‐butylpyridine (4‐TBP; 98%), bis(trifluoromethane)sulfonimide lithium salt (Li‐TFSI; 99.95%), tris(pentafluorophenyl)borane (BCF; 95%) were purchased from Sigma Aldrich. Octadecene (ODE; technical grade, 90%), methyl acetate (MeOAc; 99%), hexane (97%), octane (99%), and chlorobezene (CB; 99.8%, anhydrous) were obtained from Acros Organics. Ethyl acetate (EtOAc; ≥99.8%), toluene (99.9%), and acetonitrile (99.9%) were obtained from Thermo Fisher Scientific. 2‐Phenylethlyammonium iodide (PEAI) and FK209 Co(III) TFSI salt (FK209) were purchased from Great Cell Solar. 2,2′,7,7′‐Tetrakis[N,N‐di(4‐methoxyphenyl)amino]−9,9′‐spirobifluorene (spiro‐OMeTAD; 99.9%) was bought from Xi'an Polymer Light Technology Corp. Poly[bis(4‐phenyl)(2,4,6‐trimethylphenyl)amine] (PTAA; M_w_ 30 000) was purchased from Ossilla. Potassium bromide (KBr; 99% FTIR grade) was purchased from Specac. Indium doped tin oxide‐coated glass (ITO) was bought from Yingkou Shangneng Photoelectric material Co., Ltd. Glass substrates were produced from epredia microscope slides.

### Synthesis of Perovskite QDs

The synthesis of the perovskite QDs follows the hot injection method, first published by Protesescu et al., with several adjustments. To prepare the Cs(oleate) solution, 0.25 g of Cs_2_CO_3_, 25 mL of ODE, and 1 mL of OA were loaded in a two‐neck round‐bottom flask and degassed for 1 h at 90 °C in vacuum. After that, the flask was filled with nitrogen and heated to 150 °C until all reactants reacted and a clear solution was obtained. The Cs(oleate) was then stored in nitrogen at 70 °C until usage. For the synthesis of the CsPbI_3_, 1 g of PbI_2_ and 60 mL of ODE were filled in a three‐neck round‐bottom flask and degassed for 1 h at 120 °C in a vacuum. Subsequently, the flask was filled with nitrogen, and 6 mL of OLA and 6 mL of OA were injected simultaneously. The flask was again pumped to vacuum for 30 min until a yellow transparent solution was obtained. Then, the flask was filled with nitrogen and heated to 170 °C. At the target temperature, 8 mL of Cs(oleate) were quickly injected into the flask under vigorous stirring. The solution turned dark red, and after 7 s, the reaction was quenched with an ice‐water bath. For the purification, the as‐prepared CsPbI_3_ QDs were split in 6 centrifugation tubes and 12.5 mL of the crude solution were mixed with 37.5 mL of MeOAc in one tube and centrifuged for 10 min at 8000 rpm. The supernatant was discarded, and the wet CsPbI_3_ pellets in each centrifugation tube were re‐dispersed in 3 mL of hexane. The solution was again mixed with 5 mL of MeOAc and centrifuged for 10 min at 6000 rpm. The supernatant was removed, and the precipitates of all tubes were dispersed in 1.5 mL of octane and combined in one tube. This solution was centrifuged for 5 min at 4000 rpm, and this time, the supernatant was collected and stored overnight at 4 °C. After that, the solution was centrifuged again for 5 min at 4000 rpm, and again the supernatant was collected. The concentration of the obtained CsPbI_3_ QD dispersion was optically determined and further dispersed with octane to a concentration of 75 mg mL^−1^ for further use.

### Fabrication of CsPbI_3_ QD Thin Films

For the fabrication of thin film CsPbI_3_ QD samples, the procedure reported previously^[^ ^21^ ^]^ was followed with slight modifications. Glass (for films) or ITO substrates (for solar cells) were cleaned thoroughly by sonicating them in acetone and IPA for 15 min, respectively. The samples were then subjected to an oxygen plasma for 10 min to activate the surface and remove organic remains. For spectroscopic measurements, QD films were cast directly on the glass substrate. For solar cells, the ITO substrates were coated with a SnO_2_ film. A colloidal SnO_2_ solution of 0.15 m was prepared following the previous recipe of Yang et al.^[^ ^42^ ^]^ The layer was spin‐coated in ambient conditions at 3000 rpm and annealed at 200 °C for one hour. For the ligand exchange of the CsPbI_3_ QDs, NaOAc in MeOAc (1 mg mL^−1^) and PEAI in EtOAc (1 mg mL^−1^) were stirred overnight and filtered. For the deposition of the QD layers, the substrates were transferred to a nitrogen‐filled glovebox. The QD dispersion obtained from the synthesis was then spin‐coated dynamically on the substrates at 1000 rpm for the first 5 s and 2000 rpm for the following 10 s. To exchange the long organic ligands, the film was immersed in a solution of NaOAc in MeOAc for 15 s and was spin‐dried, followed by soaking and spin‐drying the film in MeOAc for 5 s for three times. This procedure was repeated four times to obtain a layer of approximately 250 nm. Afterward, the sample was soaked in a solution of PEAI in EtOAc for 10 s and spin‐dried. The PEAI‐treated sample was then washed with EtOAc. For the HTL, spiro‐OMeTAD was dissolved in CB at a concentration of 91.4 mg mL^−1^. To 1 mL of this solution, three dopants were added: 36.1 µL of 4‐TBP, 20.49 µL of dissolved Li‐TFSI (prepared by dissolving 522.5 mg mL^−1^ in acetonitrile), and 8.95 µL of dissolved FK209 (prepared by dissolving 375.8 mg mL^−1^ in acetonitrile). The prepared HTL was spin‐coated at 3000 rpm. After that, the devices were stored overnight in dry air to achieve oxygen doping of the spiro‐layer. For devices with doped PTAA as HTL, PTAA was dissolved in toluene (15 mg mL^−1^) and doped with 20% in weight with BCF. This solution was spin‐coated on the active layer at 3000 rpm. The devices were finalized with an 80 nm thick layer of gold deposited in a thermal evaporation process, leading to 8 devices on 1 substrate with an area of 4.5 mm^2^ per device.

### Degradation Monitoring

Degradation was carried out in a self‐built environmental rig, capable of being loaded from an N_2_ glovebox where the samples were stored. The rig's amount of nitrogen, oxygen, and humidity was monitored during measurements by a Rapidox 2100. For nitrogen‐only flows, the base content of O_2_ and H_2_O was in the low ppm. For the N_2_/O_2_ mixture, 20%–21% O_2_ was set; when humidity was introduced to the measurement chamber, 30% relative humidity (RH) was set. An Abet Sunlight Class A solar simulator provided the artificial AM1.5 sunlight at 1000 W m^−2^. Photovoltaic parameters were recorded using a Keithley 2450 source measure unit while the samples were in the respective environmental conditions and under light exposure. Both forward and backward bias directions were recorded.

### Optical Absorbance

Absorbance measurements were conducted in the air using a JASCO UV–vis spectrometer V‐770. The samples were brought to the spectrometer in air‐sealed boxes to minimize environmental exposure. Samples for absorbance measurements were prepared on glass substrates using the same fabrication method as for solar cells but without extraction layers.

### Photoluminescence

PL measurements were performed on an Edinburgh FS900 photoluminescence spectrometer. The samples were brought to the spectrometer in air‐sealed boxes to minimize environmental exposure. A 450 W xenon arc lamp was used as an excitation source. 750 nm blaze gratings were used on both excitation and emission arms. Signals were detected by a cooled Hamamatsu R2658 photomultiplier system. The emitted light was detected under an angle of 90° with respect to the excitation beam. The emission spectra were recorded after excitation at 470 nm with a dwell time of 0.2 s and were averaged over 5 scans.

### X‐Ray Diffraction

Samples for XRD measurements were prepared on glass substrates using the same fabrication method as for solar cells but without extraction layers. The samples were brought to the diffractometer in air‐sealed boxes to minimize environmental exposure. X‐ray spectra were collected on a Bruker Advance D8 diffractometer equipped with a 1.6 kW Cu‐Anode (λ = 1.54060 Å) and a 2 mm slit. All scans were performed in 1D mode using a detector from LYNXEYE_XE_T in parallel beam geometry. The scan was performed from 5° to 50°, with a step size of 0.01° and an acquisition time of 0.1 s per step. The data was background‐corrected using the Bruker Diffrac.Eva software.

### X‐Ray Photoemission Spectroscopy

XPS measurements were carried out in an ultrahigh vacuum chamber (ESCALAB 250Xi by Thermo Scientific, base pressure: 2 × 10^−10^ mbar) using an XR6 monochromated Al Kα source (hν = 1486.6 eV) and a pass energy of 20 eV. A spot size of 650 µm was utilized for the analysis. Samples for XPS measurements were prepared on ITO substrates using the same fabrication method as for solar cells but without extraction layers. For the transfer of the samples, they were exposed to air only for a short time span of approximately 30 s. All measurements were performed in the dark.

### Transmission Electron Microscopy

The transmission electron microscope (TEM) images were taken with a JEOL F200 operated at 200 kV acceleration voltage. Suspended nano‐particles were drop‐cast on a TEM copper‐grid. The grid had a thin supporting layer of a FormVar film, topped with amorphous carbon. The characterization started only after complete solvent evaporation.

### Scanning Electron Microscopy

Cross‐sectional images of the layered devices were acquired on the Gemini SEM 500 SEM from ZEISS with a landing energy of 1.5 keV and the InLens detector.

### Fourier Transform Infrared Spectroscopy

To measure Fourier Transform Infrared Spectroscopy (FTIR), three samples for each condition were prepared on glass substrates and subsequently scratched off by a spatula onto a weighing paper. The powder was mixed and ground in a mortar with 30 mg of KBr. After that, it was pressed into a pellet with a pressure of 1.7 t with a mini pellet press of 7 mm in diameter (Specac). For the background measurement, a sample with only KBr was prepared. The FTIR measurements were performed using an IRSpirit from Shimadzu, averaging 20 measurements.

## Conflict of Interest

The authors declare no conflict of interest.

## Supporting information



Supporting Information

Supporting Information

## Data Availability

The data that support the findings of this study are available from the corresponding author upon reasonable request.
