# Supplementary material for: Post‐Degradation Recovery of CsPbI3 Quantum Dot Solar Cells
Source: Small. 2025 Jan 9;21(7):2409709. doi: 10.1002/smll.202409709 (PMC11840470; doi:10.1002/smll.202409709)
Supplement: Supplementary file 1 — Supporting Information [file SMLL-21-2409709-s001.docx]

Supporting Information

Post-Degradation Recovery of CsPbI_3_ Quantum Dot Solar Cells

Julius Brunner^a),b)^, Angelika Wrzesińska-Lashkova^a),b)^, Lucas Scalon^a),c)^, Ruth Pinheiro Muniz^a)b)^, Anatol Prudnikau^b)^, Darius Pohl^d)^, Marcus Löffler^d)^, Fabian Paulus^b),e)^, Yana Vaynzof*^a),b),e)^

*Fabrication of bulk perovskite solar cells:* In the case of the regular (nip-type) architecture, we adopt a previously reported protocol^40^. The FTO-coated glass substrates (Yingkou Shangneng Photoelectric material Co., Ltd.) were cleaned with a solution of Hellmannex III (2% vol in water), acetone, and isopropanol by ultrasonication for 20 min each. The FTO glasses were dried using nitrogen and annealed in an UV-ozone chamber for 10 min. Then, the electron transport layer was deposited. A 15 wt% solution of SnO₂ in water was diluted in a 1:4 (v:v, SnO₂:deionized water) ratio with 3 mg ml^-1^ of KCl. The solution was then subjected to ultrasonication for 5 min and filtration through a 0.22 µm filter before deposition. For the deposition, 80 µl of the SnO₂:KCl solution was applied to the FTO/glass substrate and spin-coated at 3000 rpm for 30 s, using 2000 rpm s^-1^ of acceleration. This was followed by a thermal annealing at 150 °C for 30 min in air. Subsequently, the glass/FTO/SnO₂ films were subjected to a 30 min annealing process in a UV-ozone chamber, after which they were transferred to a dry box with a relative humidity of less than 5%. For the perovskite layer, a composition of Cs_0.05_FA_0.85_MA_0.10_Pb(I_0.83_Br_0.17_)_3_ was employed. The preparation of the perovskite precursor solution and its deposition were performed in accordance with previously published reports.^41^ The perovskite precursors were acquired from TCI Greatcell Solar Materials, and the solvents from Sigma Aldrich. The HTL and gold electrode were deposited the same way as mentioned above for the CsPbI_3_ QD devices.


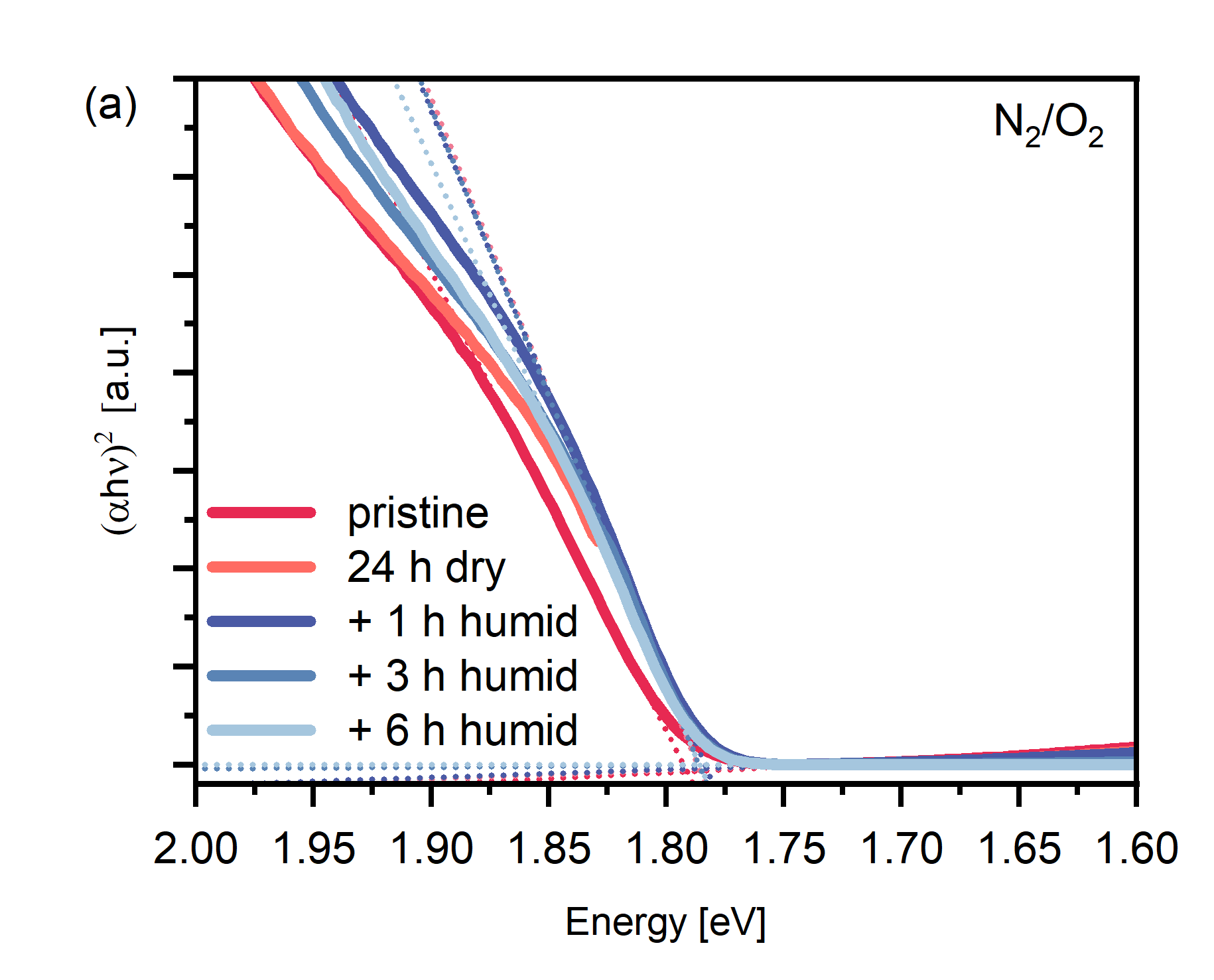

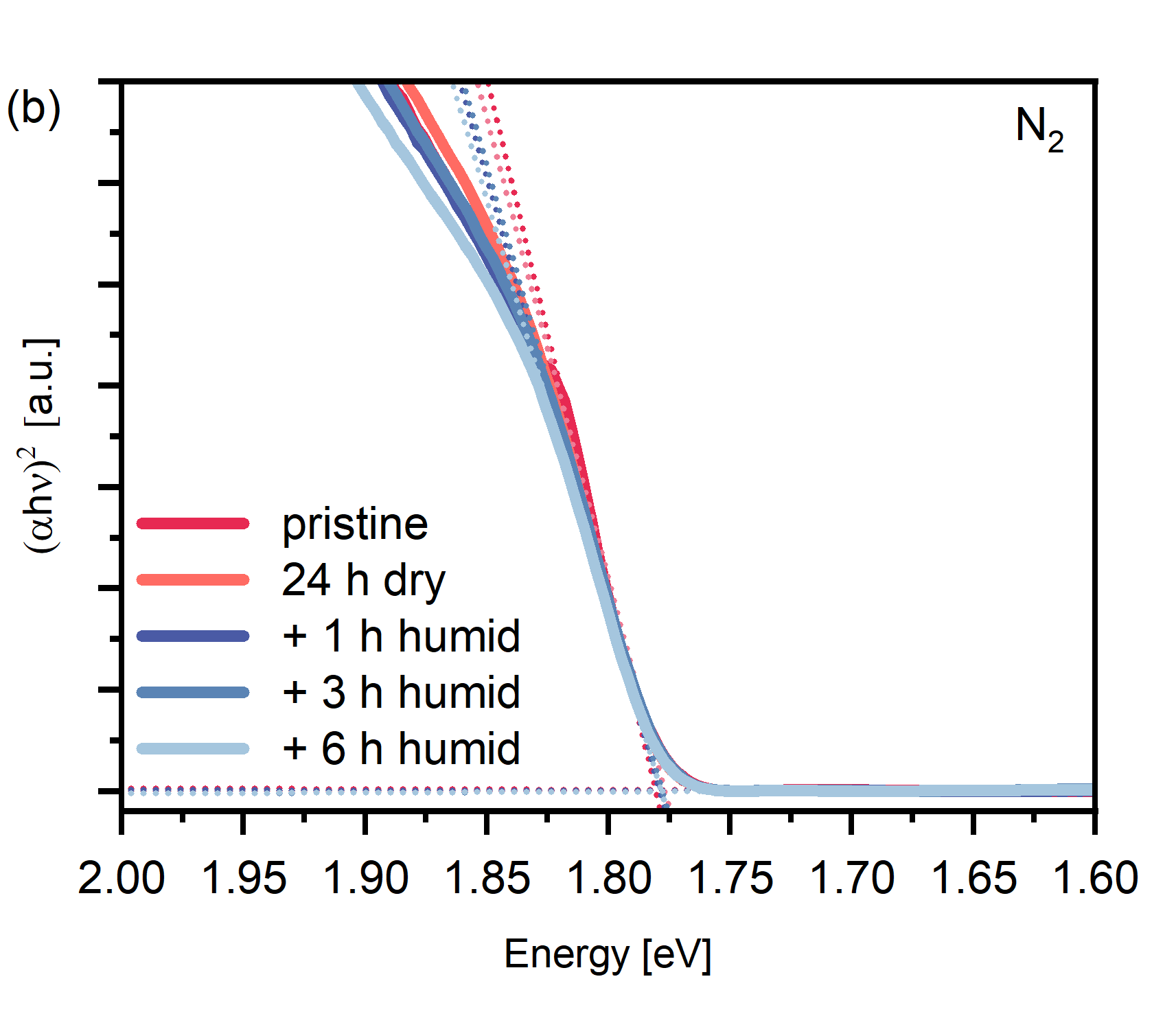


**Figure S1.** Tauc plots of CsPbI_3_ films after degradation in (a) N_2_/O_2_ and (b) N_2_ with linear fits calculated with origin.


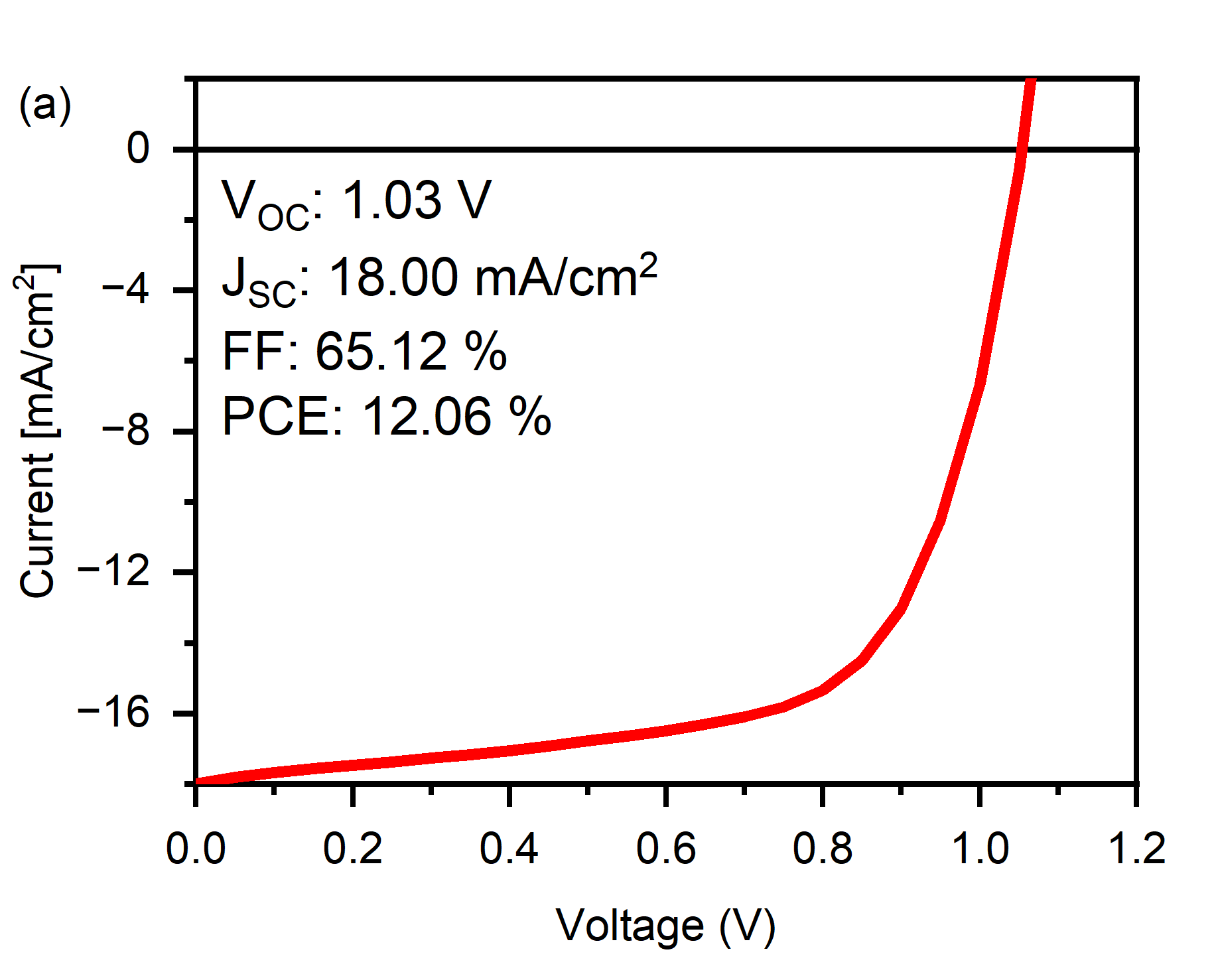

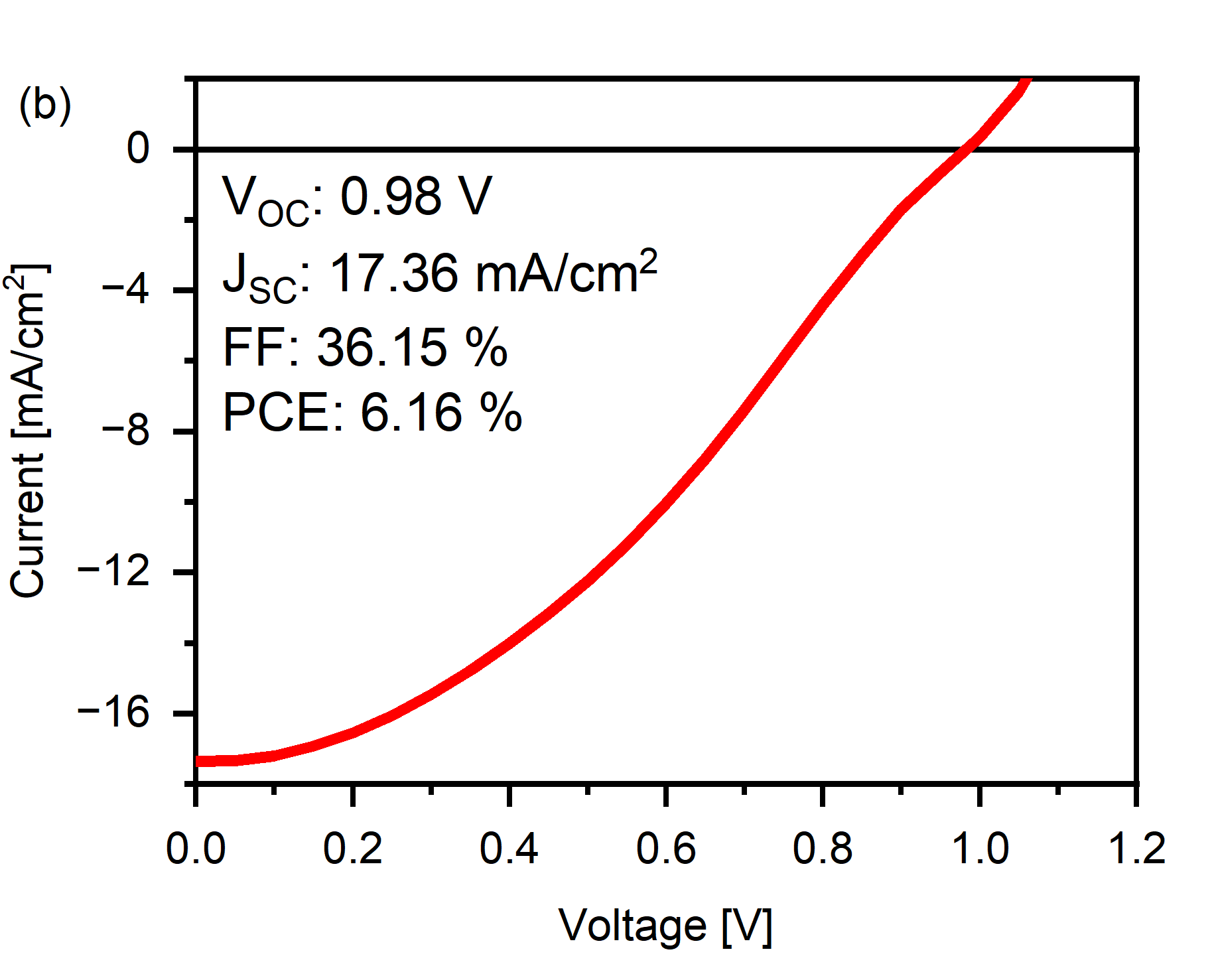
**Figure S2.** (a) J-V curve of the device with bulk perovskite instead of QDs. (b) J-V curve of the device with doped PTAA as HTL instead of spiro OMeTAD.


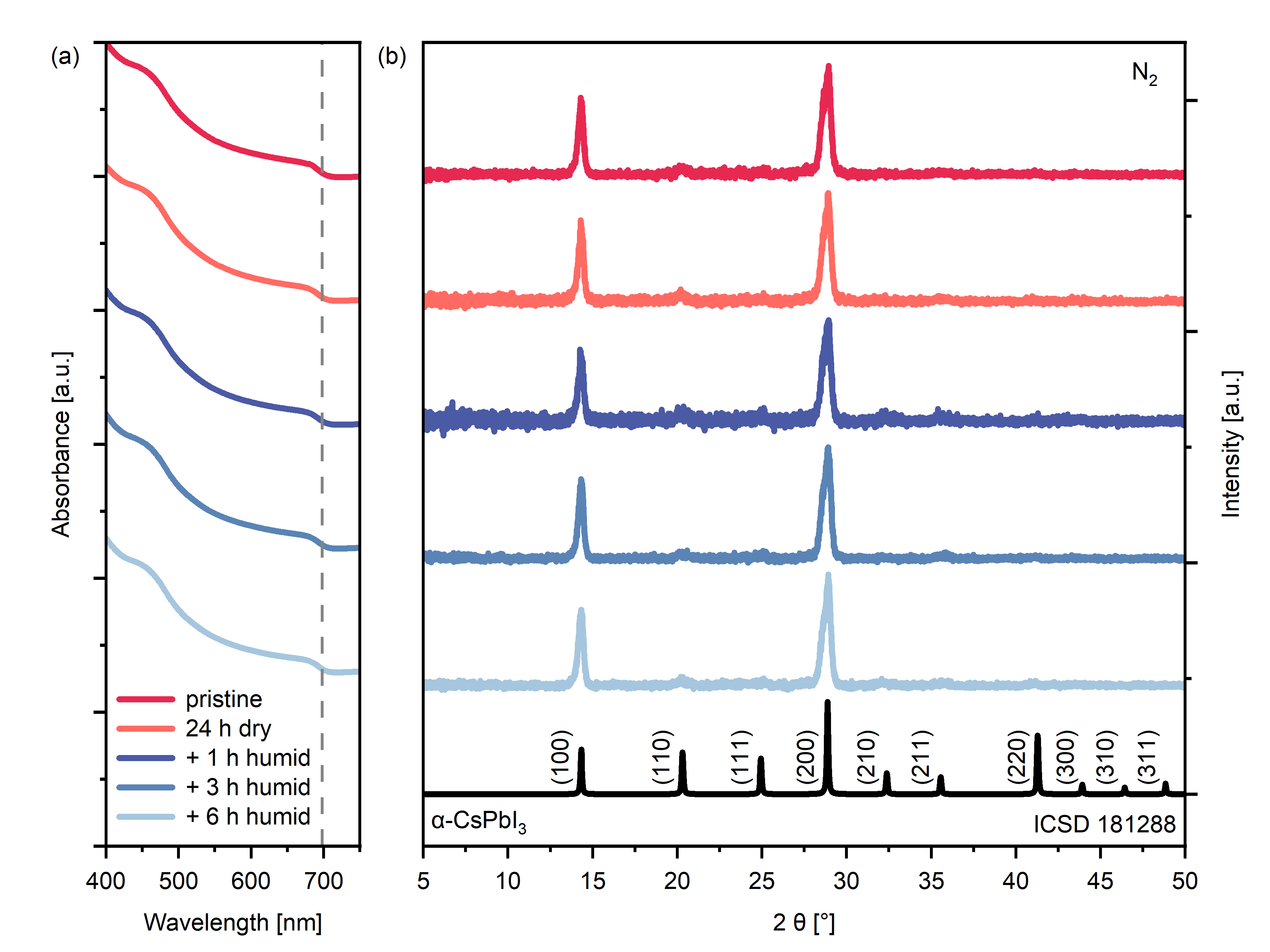
 **Figure S3.** (a) Absorbance spectra of CsPbI_3_ QD layers after exposure to dry and humid N_2_ atmosphere. The dashed line marks the bandgap calculated from Tauc plots (see Figure S1). (b) XRD pattern of the same QDs with theoretical data from the ICSD database.


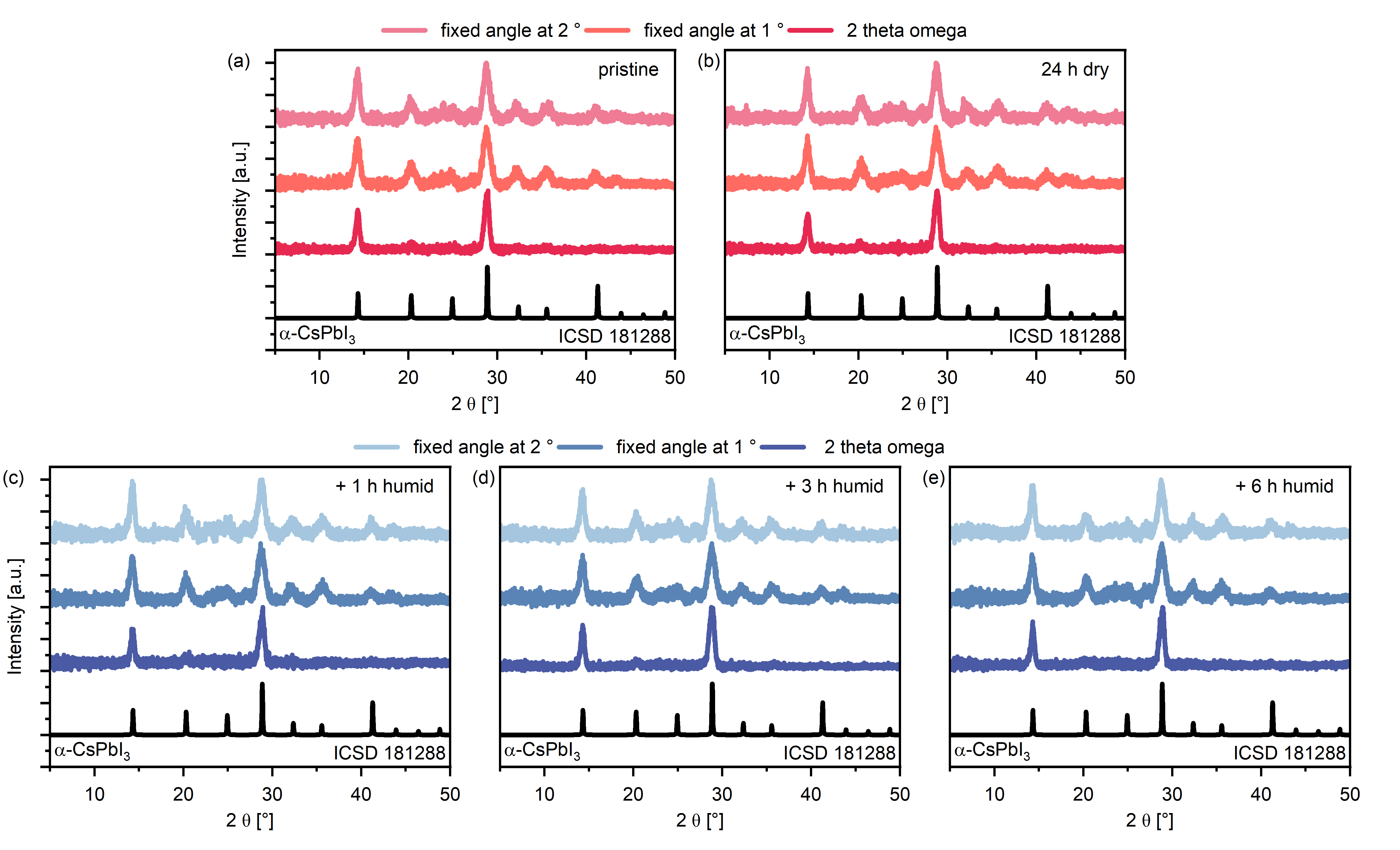
**Figure S4.** 2-theta-omega and 2-theta (fixed angle at 1° and 2°) XRD scans of samples after exposure to N_2_/O_2_ environment. (a) Pristine sample. (b) After 24 h of dry N_2_/O_2_. (c) After 24 h of dry and 1 h of humid N_2_/O_2_. (d) After 24 h of dry and 3 h of humid N_2_/O_2_. (e) After 24 h of dry and 6 h of humid N_2_/O_2_.

**
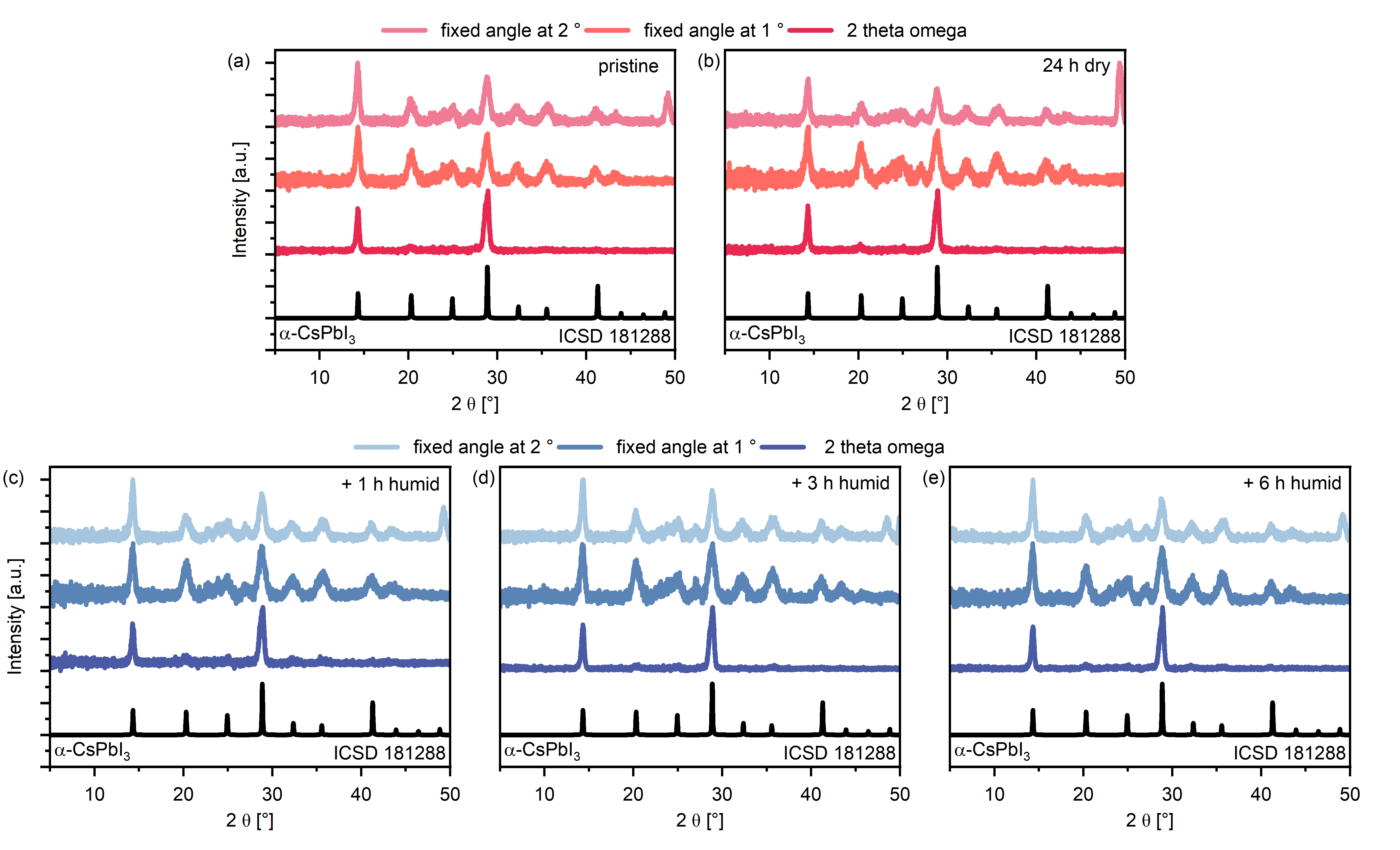
Figure S5.** 2-theta-omega and 2-theta (fixed angle at 1° and 2°) XRD scans of samples after exposure to N_2_ environment. (a) Pristine sample. (b) After 24 h of dry N_2_. (c) After 24 h of dry and 1 h of humid N_2_. (d) After 24 h of dry and 3 h of humid N_2_. (e) After 24 h of dry and 6 h of humid N_2_.


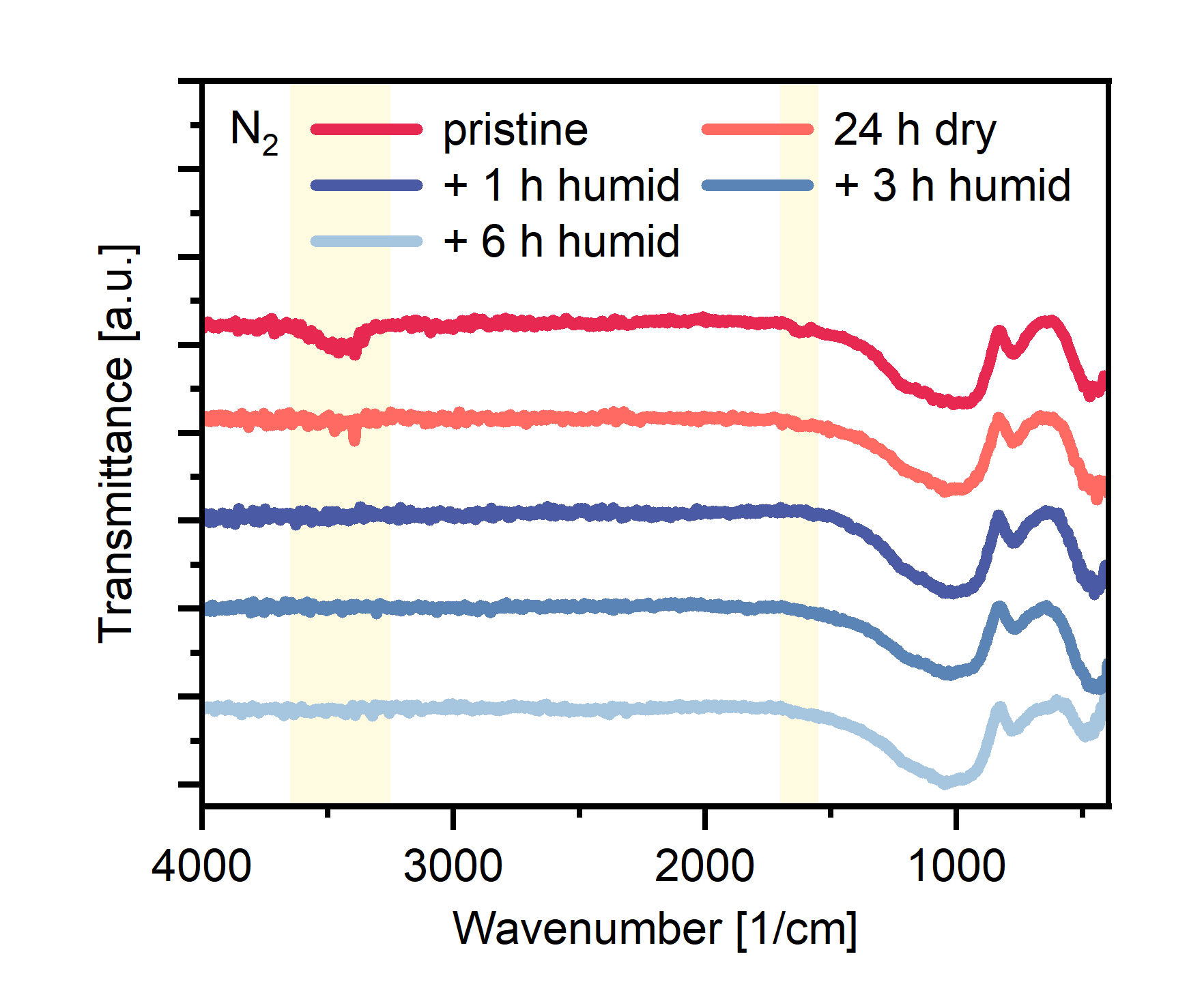


**Figure S6.** FTIR spectra of the treated samples in N_2_. The region of the H-O-H bending mode at around 1700 cm^-1^ as well as O-H stretching at around 3500 cm^-1^ are highlighted in pale yellow.


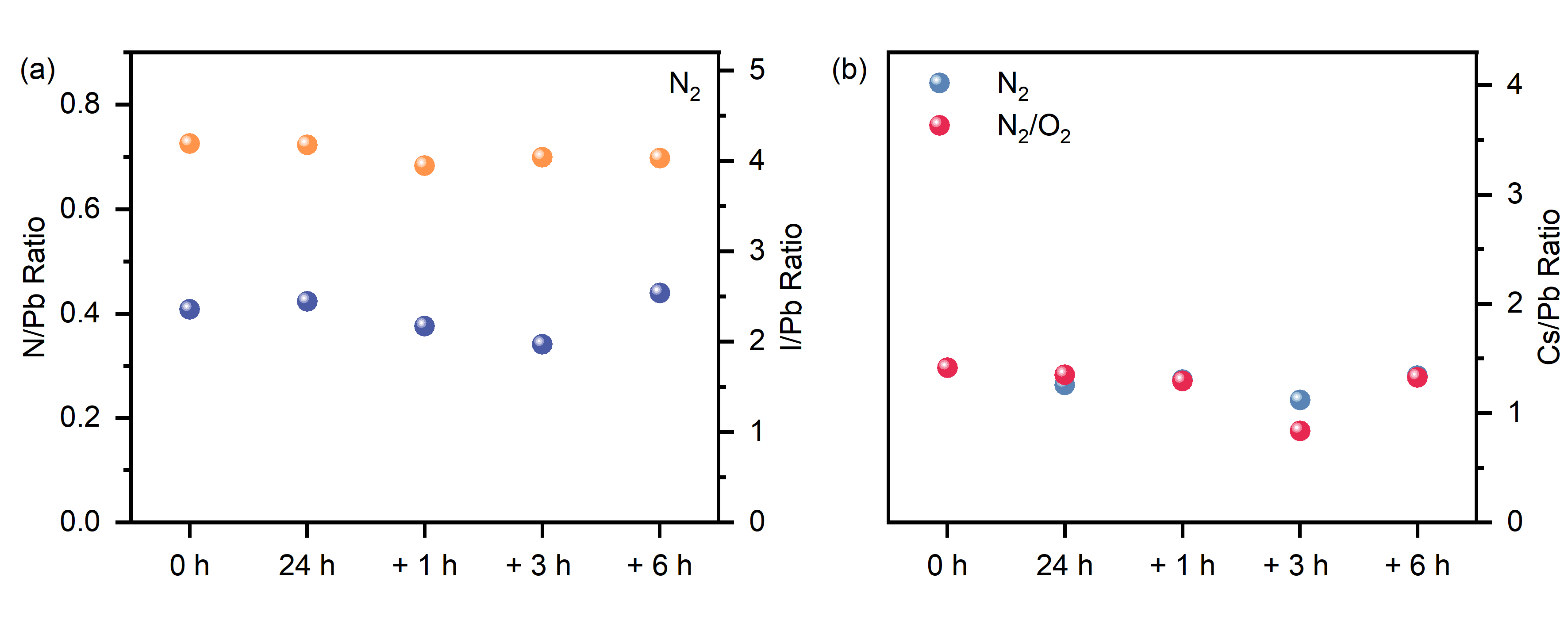


**Figure S7.** XPS of CsPbI_3_ films after degradation in dry and humid N_2_/O_2_ and N_2_ atmospheres. (a) N/Pb and I/Pb ratios in N_2_. (b) Cs/Pb ratio in N_2_ and in N_2_/O_2_.


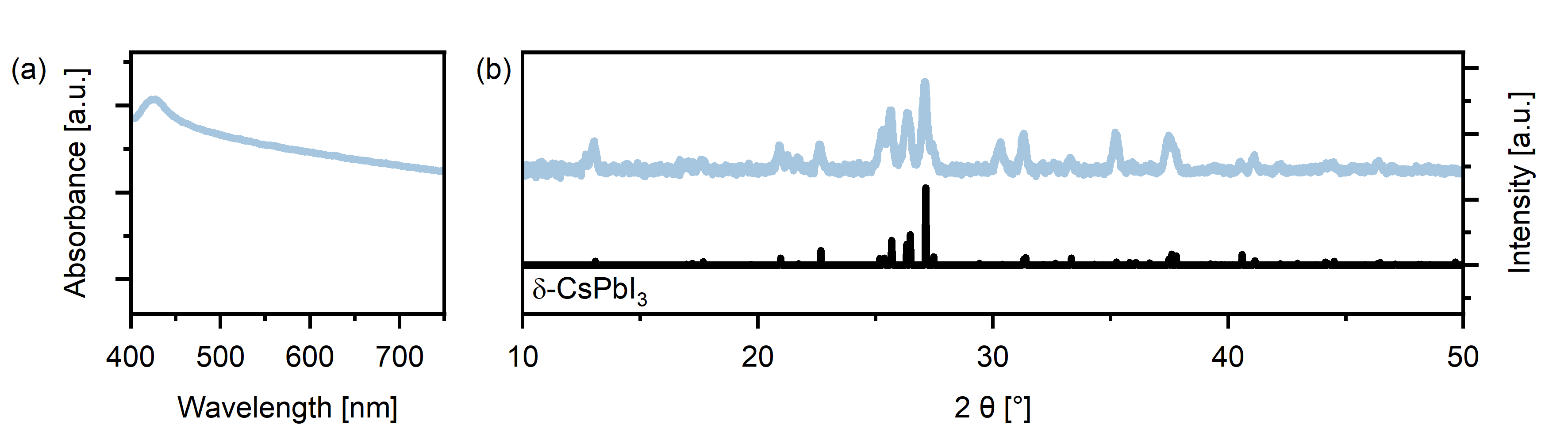


**Figure S8.** (a) Absorbance and (b) XRD pattern of CsPbI_3_ after excessive exposure to humid N_2_/O_2_ with crystal data from reference ^42^.


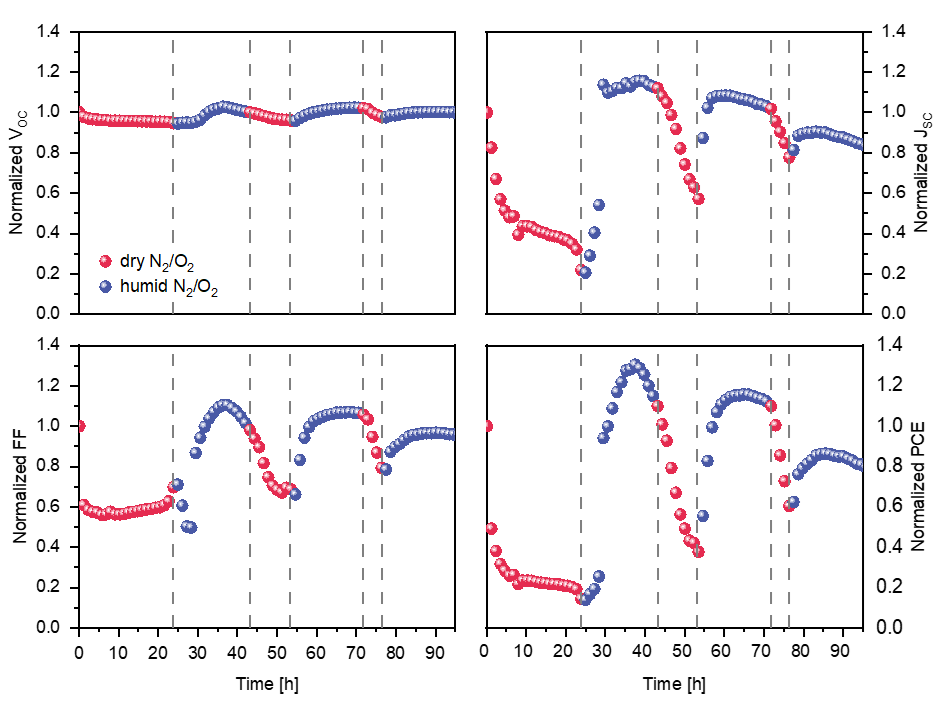


**Figure S9.** Cycles of dry and humid N_2_/O_2_ conditions. Dashed vertical reference lines indicate the change between dry and humid atmospheres.
